# Supplementary material for: The impact of macroeconomic policy uncertainty on micro-enterprise investment efficiency—Empirical evidence from China
Source: PLoS One. 2024 Jun 12;19(6):e0304667. doi: 10.1371/journal.pone.0304667 (PMC11168625; doi:10.1371/journal.pone.0304667)
Supplement: Attachment — Submitted filename: Response to Reviewers.docx [file pone.0304667.s002.docx]

**Response to Reviewers**

We would like to express our heartfelt gratitude to two anonymous reviewers for their valuable feedback. Our manuscript, titled “**The impact of macroeconomic policy uncertainty on micro-enterprise investment efficiency—Empirical evidence from China**”, benefited significantly from the constructive comments and insights from the review team. Based on the suggestions received, we have made careful revisions to the original manuscript. In the revised manuscript, all changes are marked in red. In addition, we have also carefully proofread this manuscript for typographical, grammatical, and other errors. We hope the revised manuscript can meet your standard of quality and address the concerns raised by the reviewers. In the following, you will find our detailed point-by-point responses to the reviewers’ comments.

**Reviewer #1:**

**The analysis of the impact of macroeconomic policy uncertainty on micro-enterprise investment efficiency in the article yielded meaningful conclusions. However, it is important to note that there are deficiencies in various sections of the article. Therefore, my review conclusion is that a Major Revision is required. I hope the authors can improve the article based on the following suggestions, although these revisions may not be easy to implement:**

***Response*:**

We sincerely appreciate your approval as well as valuable feedback on our manuscript. All these comments have helped us significantly in improving the quality of our manuscript. Based on your comments and suggestions, we have made extensive changes to the manuscript in the revised manuscript. Our responses to your specific comments are provided below. We also provide a copy of the relevant sections (marked in red) following our responses.

**1. The introduction section is incomplete, as it fails to clearly identify the gap in existing research and the importance of this study.**

***Response*:**

Thank you for pointing this out. Following your suggestions, in the revised manuscript, we have reorganized and revised the Introduction section in hopes that it clearly identifies the existing research gaps and the significance of this study. The specific content for this change is as follows:

*Section 1, paragraph 2*

*…*

*According to existing literature, scholars studying the impact of economic policy uncertainty on the social economy focus on the relationship between economic policy uncertainty and macro factors such as economic development, the foreign exchange market, and financial risk. Meanwhile, scholars studying the efficiency of enterprise investment pay more attention to the impact of corporate governance, the external institutional environment, and the informal system on investment efficiency. Only a few scholars have integrated economic policy uncertainty and enterprise investment efficiency into the same research framework. However, there are significant differences in the research conclusions of these scholars, which can be broadly categorized into two views. There are two theories regarding the impact of economic policy uncertainty on enterprise investment. The first is the constraint theory, which suggests that uncertainty will worsen underinvestment, leading to reduced investment efficiency. The second is the efficient decision-making theory, which posits that uncertainty will increase the prudence and rationality of investment decisions, thereby improving investment efficiency. Furthermore, the limited literature available fails to systematically examine the mechanism and heterogeneity of economic policy uncertainty that affects corporate investment efficiency. Therefore, our research holds theoretical significance and importance in three key aspects. Our research contributes to resolving differences in existing literature by providing new evidence supporting the constraint theory. Secondly, this study analyses the impact of economic policy uncertainty on enterprise investment efficiency through three factors: digital financial development, enterprise ESG performance, and entrepreneur information. The findings provide theoretical support and practical measures for enterprises to cope with economic policy uncertainty and enhance investment efficiency. Thirdly, this study identifies the varying impact of economic policy uncertainty on different types of enterprises based on their valuation level, governance structure, audit reputation, and bank-enterprise relationship. This information is valuable for enterprises to manage the uncertain impact of economic policy. The above outlines the theoretical significance and practical application of this study. Given the significant number of micro-enterprises in China, which are heavily impacted by macro policies, addressing the aforementioned issues is of utmost importance.*

**2. The rationale for focusing on China as the study's subject, especially considering PLOS is an international journal, needs to be clearly explained. Additionally, the paper lacks a comparison of its findings with those from other countries, a comparison that should be present in the introduction, literature review, or conclusion sections.**

***Response*:**

Thank you for your valuable suggestion. We sincerely apologize for not clearly explaining in the original manuscript why China was chosen as the subject of our research. Following your advice, we have clarified this reason in the revised manuscript. In addition, we reorganized and revised the conclusion, and compared our research results with those of other countries. The specific content for this change is as follows:

*Section1, paragraph 1, line 3*

*…*

*China, with its socialist market economic system, exhibits distinct policy-oriented characteristics. The economic policies crafted by the Chinese government wield a profound influence on the country's enterprises. Given China's status as the world's leader in listed companies, it offers a robust sample size for research, enhancing the credibility and applicability of the study. As such, China serves as an ideal research subject.*

*…*

*Section5, paragraph 1, line 8*

*…*

*In fact, many scholars in other countries have obtained similar research results on the impact of economic policy uncertainty on corporate entities, in addition to the above conclusions drawn by us with China as the research object. For example，Jens (2017) highlights the effect of the presidential election on firm investments, showing that investments decrease before and rebound after an election. According to Kahle & Stulz (2013), capital expenditures and corporate borrowing usually fall sharply in times of policy uncertainties and financial crises. Gulen & Ion (2015) use the EPU index to argue the impact of uncertainty on corporate decisions.*

*…*

**3. I have doubts about the key explanatory variable. Macroeconomic policy is multifaceted, so is it reasonable to measure it using only one index? Additionally, how is it measured and calculated specifically? Merely citing literature does not give me sufficient understanding of this index. Finally, have any journals published research using this index? Similarly, the selection of the key explained variable still needs literature support, as this indicator is quite indirect, as mentioned in the article.**

***Response*:**

Thank you for pointing out the problems.

We sincerely apologize for not clearly explaining the rationale behind our paper's key explanatory variable, Economic Policy Uncertainty (EPU), in the original manuscript. The academic community primarily utilizes two methods to measure EPU: one developed by Huang and Luk (2020) and the other by Baker et al. (2016), both derived from text analysis. We chose Huang and Luk's method over Baker et al.'s due to its broader data source and objectivity (Wen et al., 2022, Yu et al., 2021; Yao et al., 2021), drawing from ten mainland Chinese newspapers instead of one from Hong Kong, reflecting a more accurate situation in mainland China. For the dependent variable (Abs), we directly chose the non-efficiency investment index from the CSMAR database as the proxy for corporate investment efficiency. This decision is based on Richardson's (2006) methodology for measuring non-efficient investments using model residuals. To verify the validity of this index, we recalculated it following Richardson's method and found our results to be consistent with the CSMAR database. The specific measurement methods of explanatory variables and explained variables are as follows:

*Section3.2, paragraph 4, line 1*

*…*

*The index is measured as follows:*

*Huang Y. & Luk count the number of occurrences of articles discussing economic policy uncertainty in leading daily general interest Chinese-language newspapers. Huang Y. & Luk P. construct a monthly index starting in January 2000 by searching for relevant keywords in the electronic archives of ten newspapers: Beijing Youth Daily, Guangzhou Daily, Jiefang Daily, People's Daily Overseas Edition, Shanghai Morning Post, Southern Metropolis Daily, The Beijing News, Today Evening Post, Wen Hui Daily and Yangcheng Evening News. Huang Y. & Luk P. obtain newspaper contents and search for related keywords in the digital archives wisers Information Portal. This platform covers important and influential papers from large cities representative of the newspaper market in urban areas. For each newspaper, they search for articles which contain at least one keyword in each of the three criteria, namely (1) Economy, (2) Uncertainty, and (3) Policy. Table 1 shows the keywords in each criterion and their English translation.*

***Table1.*** *Relevant keywords for compiling China economic policy uncertainty index*

| *Criteria* | *English* |
| --- | --- |
| *(1) Economic* | *Economic/economy/financial* |
| *(2) Uncertainty* | *Uncertainty/uncertain* |
|  | *Volatile* |
|  | *Unstable/unclear* |
|  | *Unpredictable* |
| *(3) Policy* | *Policy/measures* |
|  | *Politics* |
|  | *Government/authority* |
|  | *President* |
|  | *Prime minister* |
|  | *Reform* |
|  | *Regulation* |
|  | *Fiscal* |
|  | *Tax* |
|  | *People's Bank of China/PBOC* |
|  | *Deficit* |
|  | *Interest rate* |

*In order to match the EPU index to the year level, we refer to the practice of Li et al. (2016), the annual data of the year is obtained by dividing the arithmetic average of the twelve monthly data of the year by 100.*

*…*

*Referring to Richardson (2006)’s measurement method of inefficient investment, we use model residuals to measure the level of inefficient investment of enterprises, and constructs the following regression model.*

$\boldsymbol{Inv}_{\boldsymbol{i,t}}\boldsymbol{=}\boldsymbol{\theta}_{\boldsymbol{0}}\boldsymbol{+}\boldsymbol{\theta}_{\boldsymbol{1}}\boldsymbol{Lev}_{\boldsymbol{i,t}\boldsymbol{-}\boldsymbol{1}}\boldsymbol{+}\boldsymbol{\theta}_{\boldsymbol{2}}\boldsymbol{Growth}_{\boldsymbol{i,t}\boldsymbol{-}\boldsymbol{1}}\boldsymbol{+}\boldsymbol{\theta}_{\boldsymbol{3}}\boldsymbol{Age}_{\boldsymbol{i,t}\boldsymbol{-}\boldsymbol{1}}\boldsymbol{+}\boldsymbol{\theta}_{\boldsymbol{4}}\boldsymbol{Cash}_{\boldsymbol{i,t}\boldsymbol{-}\boldsymbol{1}}\boldsymbol{+}\boldsymbol{\theta}_{\boldsymbol{5}}\boldsymbol{Return}_{\boldsymbol{i,t}\boldsymbol{-}\boldsymbol{1}}\boldsymbol{+}\boldsymbol{\theta}_{\boldsymbol{6}}\boldsymbol{Size}_{\boldsymbol{i,t}\boldsymbol{-}\boldsymbol{1}}\boldsymbol{+}\boldsymbol{\theta}_{\boldsymbol{7}}\boldsymbol{Inv}_{\boldsymbol{i,t}\boldsymbol{-}\boldsymbol{1}}\boldsymbol{+}\boldsymbol{Industry}_{\boldsymbol{i}}\boldsymbol{+}\boldsymbol{\varepsilon}_{\boldsymbol{i,t}}$	***(3)***

*Where the* ${Inv}_{it}$ *represents the new investment expenditure of the enterprise in the current period,* ${Lev}_{i,t-1}$ *represents the company’s asset-liability ratio in the previous period;* ${Growth}_{i,t-1}$ *indicates the growth rate of the company’s operating income in the previous period;* ${Age}_{i,t-1}$ *is the listing year of the previous period;* ${Cash}_{i,t-1}$ *is the cash ratio of the enterprise in the previous period, which is equal to the ratio of cash holdings to total assets at the beginning of the period;* ${Return}_{i,t-1}$ *is the stock return of the previous period;* ${Size}_{i,t-1}$ *is the size of the enterprise, expressed as the logarithm of the total assets;* ${Inv}_{i,t-1}$ *is the new investment expenditure of the enterprise in the previous period;* ${Industry}_{i}$ *represents the industry dummy variable. Next, the regression of Model (3) is carried out, and the absolute value of its residual is taken as the proxy variable of inefficient investment. The larger the absolute value is, the more serious the inefficient investment phenomenon is.*

**4. The discussion and resolution of endogeneity are insufficient. First, the specifics of the PSM operation are unclear, such as what matching methods were used and other more detailed aspects of the matching process? Second, for reporting PSM results, it is recommended to regress using the matched sample and compare it with the baseline regression results. Third, I strongly suggest the authors try using instrumental variable methods to address potential endogeneity issues (caused by reverse causality or omitted variables, etc.).**

***Response*:**

Thank you for pointing out the problems in the manuscript. We also realize that our use of PSM to deal with endogenous problems is not rigorous. Therefore, we delete the part of PSM, and accept the opinions of the reviewers, use the IV method to deal with endogenous problems. The specific contents are as follows:

*Section4.3, paragraph 1, line 1*

*…*

*4.3. Endogeneity test*

*The IV can alleviate the problem of missing variables and reverse causality to a certain extent. Therefore, this paper uses the IV method to estimate. Since the US economic policy is an important factor affecting the formulation and implementation of China’s economic policy, and the uncertainty of the US economic policy will affect the behavior of Chinese enterprises by affecting the uncertainty of China’s economic policy (Li et al., 2022). Therefore, this paper draws on the practice of Li et al. (2022), and selects the U.S. Economic Policy Uncertainty Index (UEPU) developed by Baker et al. (2016) as an exogenous instrumental variable of China’s Economic Policy Uncertainty (Epu) for testing. Of course, this indicator is also unified into an annual indicator in the same way as the EPU. To this end, we add Model (4) on the basis of Model (1), and use the two models for IV estimation:*

$\boldsymbol{UEP}\boldsymbol{U}_{\boldsymbol{t}}=\boldsymbol{\gamma}_{\boldsymbol{0}}+\boldsymbol{\gamma}_{\boldsymbol{1}}\boldsymbol{Ep}\boldsymbol{u}_{\boldsymbol{t}}+\boldsymbol{\gamma}_{\boldsymbol{2}}\boldsymbol{X}_{\boldsymbol{it}}+\boldsymbol{\eta}_{\boldsymbol{i}}+\boldsymbol{\varepsilon}_{\boldsymbol{it}}$	*(4)*

*Table 6 reports the IV estimation results of the two-stage least squares method. Among them, (1) is listed as the first stage regression, and the results show that the US economic policy uncertainty (UEPU) has a significant negative impact on China’s economic policy uncertainty (EPU), which meets the correlation requirements of instrumental variables; column (2) is the second-stage regression result. EPU has a significant positive impact on the inefficient investment of enterprises, which is consistent with the previous conclusions. The Kleibergen-Paap rk Wald F value is 9518.18, which is much larger than the Stock-Yogo threshold at the 10 % level, indicating that the risk of weak instrumental variables is very small, we chose the appropriate instrumental variables. The results of Table 6 show that our research results are still valid after using the instrumental variable method to deal with the endogeneity problem of the model.*

*Table6. Two-stage estimation results of IV*

| *Explanatory Variable* | *EPU* | *Abs* |
| --- | --- | --- |
|  | *（1）* | *（2）* |
| *EPU* |  | *0.1913****  *(2.91)* |
|  |  |  |
| *UEPU* | *-0.0854****  *(-97.56)* |  |
|  |  |  |
| *Kleibergen-Paap rk Wald F value* |  | *9518.18* |
| *Stock-Yogo critical value (10%)* |  | *16.38* |
| *Control variables* | *yes* | *yes* |
| *id Individual fixed effects* | *yes* | *yes* |
| *N* | *11022* | *11022* |

**5. Although the model setup for mechanism analysis is stated, Table 6 still needs to show the coefficients and significance levels of the mechanism variables (DFI, ESG & CON).**

***Response*:**

Thank you for pointing out this out. We sincerely apologize for not reporting the coefficients and significance levels of the mechanism variables (DFI, ESG, CON) in Table 6. In the revised manuscript (Table 7), we have included the aforementioned details, with specific modifications as follows:

*Section4.4, paragraph 4, line 1*

*…*

***Table7.*** *Mechanism test results*

| *Explanatory Variable* | *Explained variable* | | |
| --- | --- | --- | --- |
|  | *（1）* | *（2）* | *（3）* |
| *EPU* | *0.7648***  *（2.46）* | *0.7648***  *（2.46）* | *0.1472****  *（4.18）* |
| *DFI* | *0.4278*  *（1.34）* |  |  |
| *EPU*DFI* | *-0.182***  *（-2.42）* |  |  |
| *ESG* |  | *0.0364***  *（3.21）* |  |
| *EPU*ESG* |  | *-0.0276****  *（-3.19）* |  |
| *CON* |  |  | *0.155****  *（4.13）* |
| *EPU*CON* |  |  | *-0.119****  *(-4.20)* |
| *Control variables* | *Yes* | *Yes* | *Yes* |
| *id Individual fixed effects* | *Yes* | *Yes* | *Yes* |
| *N* | *11022* | *11022* | *11022* |

**6. Any heterogeneity analysis must have a theoretical basis, i.e., why such heterogeneity analysis is conducted. Additionally, the explanation of the results of the heterogeneity analysis part needs support from relevant literature.**

***Response*:**

Thank you for highlighting this issue; we greatly appreciate it. In the revised manuscript, we have reorganized and revised the content of heterogeneity analysis, hoping to explain the reasons why we choose the four perspectives of enterprise valuation, governance structure, audit reputation and bank-enterprise connection to carry out heterogeneity analysis. At the same time, we also combed the relevant literature to provide support for our interpretation of the heterogeneity test results. The details are as follows:

*Section4.5, paragraph 1, line 1*

*…*

*Based on the previous research, we will focus on the heterogeneous impact of economic policy uncertainty on different types of enterprises. Of course, this heterogeneity analysis is mainly carried out in the context of placing enterprises in the context of economic policy fluctuations. The first is the enterprise valuation. In the face of drastic fluctuations in economic policy, the difference in valuation level may make the investment decisions of enterprises have a greater difference. The second is the governance structure. The difference in governance structure will also lead to different coping strategies for enterprises to deal with economic policy uncertainty. Obviously, the risk appetite and interest demand of shareholder-controlled enterprises and manager-controlled enterprises are different. The third is audit reputation. Considering that the financial management of enterprises will have a significant impact on corporate investment, it is necessary to study the heterogeneity of enterprise samples with different audit reputations. The fourth is bank-enterprise linkage. Since bank loans are still the main source of funds for Chinese enterprises, we believe that it is necessary to examine the differences in investment efficiency of different enterprises in response to uncertainty shocks from the perspective of bank-enterprise linkage.*

*Section4.5, paragraph 2, line 7*

*…*

*According to the research of Gu & Zhu (2021): on the one hand, because the Tobin Q value reflects the future development trend of the enterprise, there are many investment opportunities, and the enterprise will carefully examine the investment plan and pay attention to the improvement of investment efficiency. On the other hand, the high valuation of enterprises indicates that investors in the market have high recognition of enterprises and low degree of information asymmetry. When faced with financing constraints, it is easier for enterprises to raise funds and alleviate underinvestment.*

*Section4.5, paragraph3, line 8*

*…*

*The equity of shareholder-controlled enterprises is highly concentrated, and the major shareholders control the absolute control of the enterprise. They often over-intervene in the management and decision-making of the enterprise, making the enterprise make more irrational investment decisions in the face of high economic policy uncertainty. Reduce investment efficiency (Gu & Zhu, 2021). Manager-controlled enterprises have a higher degree of separation of two rights. Professional managers ' more professional quality and more rational decision-making can improve governance efficiency and investment efficiency.*

*Section4.5, paragraph4, line 5*

*…*

*According to the research of Gu & Zhu (2021): the possible explanation is that the quality of accounting report information of enterprises with low audit reputation is low. In the case of economic policy uncertainty, investors reduce their investment willingness and investment scale to avoid information asymmetry, which aggravates the financing constraints of enterprises and reduces the investment efficiency of enterprises.*

*Section4.5, paragraph5, line9*

*…*

*According to the research of Zhai et al., (2014): The possible explanation is that enterprises with bank connections have better financing advantages and resources, which can effectively alleviate the financing constraints caused by EPU to enterprises, so as to maintain higher investment efficiency. The financing cost of enterprises without bank connection is higher and the financing is more difficult, so it is difficult to eliminate the impact of EPU on investment efficiency.*

**7. From the results of the entire text, it is confusing which time dimension the regression is conducted on. For example, in the model setup section, the author mentioned the time dimension is on an annual basis (*year basis*). However, some key variables are measured on a monthly and quarterly basis, and it is not detailed how the final indicators are calculated or summed to a fixed time dimension.**

***Response*:**

Thank you for pointing this out. We are so sorry for the vague expression of the time dimension in the paper. We carefully examine and modify the variable description of the paper, and unify the two non-annual indicators of EPU (explanatory variable) and entrepreneur confidence (mechanism variable) into annual indicators, and explain the specific practices in detail and clearly. The specific content is as follows:

*Section3.2, paragraph6, line1*

*…*

*In order to match the EPU index to the year level, we refer to the practice of Li et al. (2016), the annual data of the year is obtained by dividing the arithmetic average of the twelve monthly data of the year by 100.*

*…*

*This paper uses the entrepreneur confidence index compiled by the CSMAR database to measure entrepreneur confidence, Since the index is quarterly data, in order to unify it into annual data, we refer to the practice of Dong (2021), and take the natural logarithm of the arithmetic mean of the four quarterly indicators each year as the annual indicator of the year.*

**8. The conclusion section is very weak; merely summarizing the results of the entire text is insufficient. I suggest adding some necessary discussions, which could be policy implications or comparisons with the conclusions of existing related research.**

***Response*:**

Thank you for pointing this out. According to yuor suggestion, we have reorganized and revised the conclusion part, increased the policy implications of our research conclusions, and compared with existing related research. The details are as follows:

*Section5, paragraph1, line8*

*…*

*In fact, many scholars in other countries have obtained similar research results on the impact of economic policy uncertainty on corporate entities, in addition to the above conclusions drawn by us with China as the research object. For example，Jens (2017) highlights the effect of the presidential election on firm investments, showing that investments decrease before and rebound after an election. According to Kahle & Stulz (2013), capital expenditures and corporate borrowing usually fall sharply in times of policy uncertainties and financial crises. Gulen & Ion (2015) use the EPU index to argue the impact of uncertainty on corporate decisions.*

*Section5, paragraph2, line1*

*…*

*In addition, our findings also have the following policy implications: 5.1 Improve the stability and pertinence of economic policies. First, we should pay more attention to consistency and coherence in policy formulation, pay more attention to transparency and timeliness in policy release, and give micro-subjects stable policy expectations. Second, it is necessary to establish an in-depth policy effect evaluation and dynamic adjustment mechanism to improve the effectiveness of economic policies. Third, we should optimize the business environment, actively guide public opinion, respect entrepreneurship, and encourage entrepreneurs to innovate. 5.2 Vigorously promote the development of digital finance. First, it is necessary to broaden the use of digital finance, optimize the digital financial market mechanism, expand the coverage of digital financial services, and ease corporate financing constraints. Second, it is necessary to speed up the iterative upgrading of digital financial technology and guide the rational and efficient flow of key elements such as digital financial technology and digital financial talents. Third, it is necessary to encourage the extensive use and innovative application of digital technology by micro-enterprises and financial institutions to alleviate the information asymmetry and moral hazard problems in the credit market. 5.3 Improve the corporate governance structure. First, we should actively adjust the corporate governance system, improve the quality of internal control, balance ownership and management rights, and improve the shareholder restraint mechanism. The second is to strengthen the construction of enterprise ESG, actively assume ESG responsibilities, scientifically formulate ESG strategic planning, and improve the quality of ESG information disclosure. The third is to improve the quality of accounting information, improve the comprehensive quality of enterprise financial management personnel, strengthen the supervision of accounting basic work, and enhance the audit reputation of the enterprise itself. Fourth, moderately strengthen the relationship between banks and enterprises. On the one hand, it is possible to strengthen equity cooperation with financial structures such as commercial banks to strengthen equity linkages; on the other hand, we should actively introduce management talents with bank background and strengthen the personnel connection with banks.*

**Reviewer #2:**

**1. The English writing should be improved.**

***Response*:**

Thank you for pointing this out. We sincerely apologize for the lack of professionalism in the English writing of our paper. In the revised manuscript, we have polished the English expression throughout the text.

**2. Could you provide the results of sample balance test?**

***Response*:**

Of course, here are the results of the sample balance for this study.

**3. Do you consider IV to deal with endogeneity problem?**

***Response*:**

Thank you for your kind reminder. Following your advice, we've carefully considered and decided to use an Instrumental Variable (IV) approach to address the endogeneity in our paper. Your reminder has been incredibly helpful to us. The details are as follows:

*Section4.3, paragraph1, line1*

*…*

*4.3. Endogeneity test*

*The IV can alleviate the problem of missing variables and reverse causality to a certain extent. Therefore, this paper uses the IV method to estimate. Since the US economic policy is an important factor affecting the formulation and implementation of China’s economic policy, and the uncertainty of the US economic policy will affect the behavior of Chinese enterprises by affecting the uncertainty of China’s economic policy (Li et al., 2022). Therefore, this paper draws on the practice of Li et al. (2022), and selects the U.S. Economic Policy Uncertainty Index (UEPU) developed by Baker et al. (2016) as an exogenous instrumental variable of China’s Economic Policy Uncertainty (Epu) for testing. Of course, this indicator is also unified into an annual indicator in the same way as the EPU. To this end, we add Model (4) on the basis of Model (1), and use the two models for IV estimation:*

$\boldsymbol{UEP}\boldsymbol{U}_{\boldsymbol{t}}=\boldsymbol{\gamma}_{\boldsymbol{0}}+\boldsymbol{\gamma}_{\boldsymbol{1}}\boldsymbol{Ep}\boldsymbol{u}_{\boldsymbol{t}}+\boldsymbol{\gamma}_{\boldsymbol{2}}\boldsymbol{X}_{\boldsymbol{it}}+\boldsymbol{\eta}_{\boldsymbol{i}}+\boldsymbol{\varepsilon}_{\boldsymbol{it}}$	*(4)*

*Table 6 reports the IV estimation results of the two-stage least squares method. Among them, (1) is listed as the first stage regression, and the results show that the US economic policy uncertainty (UEPU) has a significant negative impact on China’s economic policy uncertainty (EPU), which meets the correlation requirements of instrumental variables; column (2) is the second-stage regression result. EPU has a significant positive impact on the inefficient investment of enterprises, which is consistent with the previous conclusions. The Kleibergen-Paap rk Wald F value is 9518.18, which is much larger than the Stock-Yogo threshold at the 10 % level, indicating that the risk of weak instrumental variables is very small, we chose the appropriate instrumental variables. The results of Table 6 show that our research results are still valid after using the instrumental variable method to deal with the endogeneity problem of the model.*

*Table6. Two-stage estimation results of IV*

| *Explanatory Variable* | *EPU* | *Abs* |
| --- | --- | --- |
|  | *（1）* | *（2）* |
| *EPU* |  | *0.1913****  *(2.91)* |
|  |  |  |
| *UEPU* | *-0.0854****  *(-97.56)* |  |
|  |  |  |
| *Kleibergen-Paap rk Wald F value* |  | *9518.18* |
| *Stock-Yogo critical value (10%)* |  | *16.38* |
| *Control variables* | *yes* | *yes* |
| *id Individual fixed effects* | *yes* | *yes* |
| *N* | *11022* | *11022* |

**4. Do you consider the unobserved factors varying with time?**

***Response*:**

Thank you for your kind reminder. We have indeed considered this issue. However, due to the problem of multicollinearity (economic policy uncertainty and annual dummy variables), we decided not to control for time-fixed effects in our baseline regression. As you mentioned, to account for potentially unobserved factors that vary over time, we followed the approach of Li et al. (2022) as closely as possible, controlling for more factors at the firm and industry levels that could affect the regression results, to mitigate the impact of unobserved time-varying factors, The specific practice is as follows:

*Section4.2, paragraph1, line1*

*…*

*4.2. Robustness test*

*Because the economic policy uncertainty index is monthly data, this paper refers to the method of Li et al. (2022), and takes the data of the twelfth month of each year (EPU-dec) as the proxy variable of EPU for regression. In addition, this paper also uses the fiscal policy uncertainty index (FEPU) and monetary policy uncertainty index (MEPU) of mainland China developed by Huang Y. & Luk P. (2019) as new explanatory variables for regression. The results are significantly positive as shown in column (3), column (4) and column (5) of table 5. Then, the control variables are adjusted. Firstly, the control variables Roa and Board are replaced by return on equity (Roe), duality (Dua) and the proportion of independent directors (Pid). In addition, in view of the fact that China’s micro-enterprise financing is still dominated by bank credit, this paper increases the two control variables of banking competition (Bank) and financing constraints (Sa) for regression.*
